# Gut Microbiota-Mediated Inflammation and Tumorigenesis: Implications for Tumor Microenvironment and Therapeutic Response

**DOI:** 10.3390/biology15212043

**Published:** 2026-11-09

**Authors:** Karthika Rajan, Vidhya Jagadish, Aravind Madhavan, Pradeesh Babu, Geetha B. Kumar, Bipin G. Nair

**Affiliations:** School of Biotechnology, Amrita Vishwa Vidyapeetham, Amritapuri, Kollam 690525, Kerala, India

**Keywords:** gut microbiota, metabolites, inflammation, tumor microenvironment, iron metabolism, therapy resistance

## Abstract

The gut microbiota is an essential component required for sustaining a normal physiological balance. An imbalanced gut flora, also known as dysbiosis, has been implicated in cancer development and progression. Current studies emphasize that changes in microbial composition can promote tumor formation through a network of interconnected signaling pathways. These include prolonged inflammatory signaling, release of toxins from microbial species, and the production of genotoxic compounds that can damage host DNA and enhance mutations. Several pathways are involved in mediating these effects, such as signaling pathways involving inflammatory mediators like cytokines, oxidative stress, and transcription factors like NF-κB. These pathways individually and collectively can contribute to a tumor-supportive environment. Apart from the direct damage caused by the microbiota, they also play a major role in the modulation of the tumor microenvironment (TME), wherein they regulate the functioning of immune cells and also affect tumor interaction with surrounding tissues. Metabolites and microbial components can modulate differentiation of immune cells, their activation, and function, thereby shaping the tumor microenvironment. Dysbiosis has been associated with impaired immune surveillance and metabolic activity, and also influences the efficacy of cancer therapies. These findings emphasize the importance of microbial–immune interactions as key determinants of cancer progression. Altogether, the gut microbiota and their metabolites regulate the tumorigenesis process, and understanding their role provides important insights into TME interactions and cancer progression. This review summarizes the major pathways and mechanisms through which the gut microbiota and their metabolites contribute to tumorigenesis, with emphasis on inflammatory signaling pathways, microbial-derived toxic factors, iron metabolism and the interactions between the microbiota and the tumor microenvironment. Additionally, we discuss current therapeutic strategies aimed at enhancing the effectiveness of cancer treatment through modulation of gut microbiota, along with their potential applications in treatment strategies and as diagnostic biomarkers.

## Simple Summary

The human gut is composed of a vast number of microorganisms that play a major role in regulating the immune system. Increasing evidence suggests that changes in these gut microbiotas and the molecules produced by them can also influence cancer development and treatment responses. This review explores how gut microbiota may contribute to cancer by promoting chronic inflammation, altering immune responses, and affecting the metabolism of nutrients and metabolites within the body. Microbial metabolites, such as short-chain fatty acids, bile acids, tryptophan metabolites, polyamines, and lipid-derived molecules, can affect both tumor cells and immune cells in the tumor microenvironment. Regulation of nutrient and metabolite metabolism by the gut microbiota can influence the tumor microenvironment, thereby modulating immune responses, inflammation, and tumor progression. The review also emphasizes the emerging relationship between iron metabolism, gut microbiota, and immune regulation, which may provide an important connection between microbial activity, tumor progression, and therapeutic response. However, the effects of microbiota are not always beneficial or harmful and can vary depending on cancer type, disease stage, microbial composition, diet, and treatment. By bringing these interconnected mechanisms together, the review provides a clearer understanding of how the gut microbiota influence cancer progression and treatment response and also emphasizes the need for developing personalized microbiota-based strategies for cancer treatment.

## 1. Introduction

### 1.1. Gut Microbiome Composition and Functional Organization

The human gut microbiome consists of a diverse community of microorganisms found in the gastrointestinal tract and these microorganisms play a fundamental role in maintaining host physiology and health [1]. This complex microbial ecosystem is established early in life and is influenced by different factors such as mode of birth, dietary patterns, antibiotic exposure, lifestyle, and host genetic constitution [2,3,4]. Advances in high-throughput sequencing technologies and metagenomic approaches have demonstrated that the gut microbiome is a highly dynamic system that continuously interacts and evolves in response to host physiology and external environmental influences [5,6]. The gut microbiome is predominantly composed of bacteria, along with significant populations of archaea, viruses, primarily bacteriophages, and eukaryotic microorganisms such as fungi and protozoa [4,7]. The gut virome, composed mainly of bacteriophages, modulates bacterial population and gene exchange [8], while the less abundant component of the eukaryotic system, fungi and protozoa, contributes to immune regulation and gut homeostasis [9].

### 1.2. The Gut Microbiome–Host Interaction: Integrating Immune, Metabolic Crosstalk

Multifaceted interactions between the gut microbiome and the human host occur through highly complex metabolic pathways that are closely linked to immune regulation and neuroendocrine communication that further influence host health and disease state [10]. The gut microbiome plays a central role in host physiology by being a metabolically active organ contributing to the conversion of dietary substrates into bioactive compounds that have an effect on multiple organ systems [11]. The fermentation of undigested dietary fibers into short-chain fatty acids (SCFAs) by the gut microbiome is one of the most significant metabolic functions of the gut, as these SCFAs comprise metabolites such as propionate, acetate, and butyrate. These metabolites play an essential role in maintaining gut homeostasis by serving as an energy source for colonocytes, regulating epithelial cell proliferation and differentiation, and maintaining intestinal barrier integrity [12,13]. Apart from the local gut-associated effects, SCFAs are also involved in systemic functions influencing lipid and glucose metabolism, leading to improved insulin sensitivity and appetite regulation through endocrine pathways [14]. The anti-inflammatory properties exerted by SCFAs are mediated through the inhibition of histone deacetylases, enzymes that remove acetyl groups from histones, leading to reduced gene expression. This will lead to immune cell differentiation and cytokine production [15].

Commensal microorganisms in the gut play a major role in promoting host nutritional and immune homeostasis. They synthesize essential micronutrients, including the B complex vitamins such as riboflavin (vitamin B2), cobalamin (vitamin B12), and folate (vitamin B9), which are required for host nutritional homeostasis and, in turn, support the growth of beneficial microorganisms and limit pathogenic microbes [16]. These commensal microorganisms are fundamental for the maturation and tolerance of the host’s innate and adaptive immune system [17]. In addition, they compete with pathogens for nutrients and epithelial attachment sites, thereby stimulating host defense mechanisms including the secretion of antimicrobial peptides and secretory immunoglobulin A (IgA) [18]. On the contrary, a disrupted normal microbial flora, also referred to as dysbiosis, can lead to impaired micronutrient metabolism and compromised immune homeostasis, and is associated with the development of numerous diseases [19].

### 1.3. Gut Microbiome Dysbiosis and Disease Progression

The systemic effect of gut microbiota imbalance is observed not only in gastrointestinal disorders but also in metabolic disorders such as type-2 diabetes, obesity, and non-alcoholic fatty liver. The imbalance affects energy harvest and contributes to systemic inflammation [20]. Several immune-mediated diseases, including asthma, allergies, and autoimmune disorders, are associated with inflammatory response due to alterations in the gut microbiome [21]. Studies have also demonstrated the interconnection between gut dysbiosis and neurologic conditions, highlighting the influence of the gut microbiome along the gut–brain axis. These affect host behavior and cognitive function through neural signaling by microbe-derived metabolites, leading to the production of immune mediators [22,23].

Altered microbial diversity and reduced abundance of commensal bacteria in the gut leads to gastrointestinal disorders such as inflammatory bowel disease, irritable bowel syndrome, and colorectal cancer [24,25]. The enhanced growth of pro-inflammatory microorganisms result in chronic inflammation in intestinal cells. This will cause impaired epithelial barrier integrity by sustained immune activation and inflammatory cytokine production, creating a tumor-promoting environment [26,27]. Certain pathogenic bacteria, such as *Fusobacterium nucleatum* is observed to play a role in carcinogenesis by inducing DNA damage, activating oncogenic signaling pathways, and suppressing antitumor immune responses [28]. Furthermore, imbalance in microbial flora contributes to altered metabolic activity that results in reduction in synthesis of beneficial metabolites such as SCFAs while enhancing the production of carcinogenic secondary bile acids promoting tumor initiation and progression [29]. Although these mechanisms are predominantly observed in colorectal cancer, they also influence systemic malignancies and response to cancer treatments such as chemotherapy, radiotherapy and immunotherapy, highlighting the potential of microbiome as a therapeutic target for immunomodulation and cancer prevention [28,29,30]. In conclusion, the development of immune system and maintenance of immune tolerance requires a diverse and healthy microbiome; disruption to this homeostasis may result in dysregulated inflammatory responses. Exploring the interaction between gut microorganisms in regulating the host physiology and disease progression presents a significant potential for developing microbiome-based diagnostic and therapeutic strategies [31].

Even though the term dysbiosis is commonly used to describe changes in the composition and functional activity of the gut microbiota associated with disease, it should not be considered a universally defined biological state or entity. Its definition differs based on the disease state, as microbial profiles corresponding to pathological conditions such as inflammatory bowel disease, obesity, diabetes, and colorectal cancer differ to a great extent; additionally, the dysbiosis is influenced by host-specific factors, such as genetics, age, lifestyle, diet, medications used, and immune status, as well as geographical variations that mold the baseline gut microbiota [19,32]. Further variability in microbiome profiles arise from the differences in analytical methodologies used for sample collection, DNA extraction, sequencing platforms, bioinformatic pipelines, and functional analyses across studies [33]. Henceforth, dysbiosis should be viewed within a specific biological, clinical, and methodological context rather than being regarded as a universally defined condition.

This review highlights the influence of gut microbiome in triggering chronic inflammation that can promote tumor initiation, progression and therapeutic responses in host cells through the interconnected mechanisms of inflammation, immune regulation, and metabolic reprogramming within the tumor microenvironment (TME). Even though these aspects have been discussed individually or in combination in previous studies [25,34,35,36], this review summarizes current evidence into a comprehensive framework that illustrates how gut microbiota-driven inflammatory, immune, and metabolic networks interact to remodel the tumor microenvironment and influence therapeutic outcomes. Instead of emphasizing individual microbial species or metabolite groups, this review underscores the dynamic crosstalk between microbial metabolites, immune cells, and the tumor microenvironment, focusing on how these interactions converge to determine disease progression and treatment outcomes. Further, it evaluates the current evidence from in vitro, animal, and human studies, discusses context-dependent effects of microbial metabolites, and identifies key translational challenges and future opportunities for microbiota-targeted precision oncology.

## 2. Immune Regulations by Gut Microbiome: From Microbes to Metabolites

Gut microbiome plays a crucial role in shaping the innate and adaptive immunity by regulating immune cell development, activation and tolerance. Dysbiosis creates a disrupted immune homeostasis leading to persistent inflammation, autoimmunity and increased susceptibility to infections such as cancer. Multiple mechanisms are employed by the gut microbiome to regulate the host immune system; firstly, in the development and priming of immune cells by shaping the maturation of gut-associated lymphoid tissue (GALT) and directing the differentiation of immune cells. The microbial impact on regulatory T cells, Treg/Th17 balance is crucial for preventing chronic inflammatory disorders. Commensal-derived signals facilitate the expansion and functional maintenance of Tregs thereby promoting immune tolerance and preventing excessive inflammatory responses. Certain members of *Clostridium* species, clusters (IV and XIVa), as well as polysaccharide A from *Bacteroides fragilis*, have been shown to promote differentiation and maintenance of Treg responses [37,38]. In addition, metabolites produced by the gut microbiota, particularly short-chain fatty acids (SCFAs), directly regulate T cell differentiation through activating G-protein-coupled receptors and inhibiting histone deacetylases enzyme leading to enhanced expression of transcription factors such as Foxp3 in regulatory T cells [39]. Foxp3 is responsible for regulating the development, stability, and function of Treg cells. Secondly, in the context of barrier maintenance and antimicrobial defense, the structural and functional integrity of the intestinal epithelium is actively supported by the commensal microorganism. Metabolites such as SCFAs promote goblet cell mucus production, upregulate tight junction protein expression and stimulate epithelial cells to secrete antimicrobial peptides such as defensins and RegIIIγ, thereby limiting pathogen colonization and translocation [40,41]. These interactions enhance mucosal barrier integrity and maintain a regulated host–microbiota interface. Recognition of microbial ligands by pattern-recognition receptors of the epithelial cells stimulate production of cytokines that further enhance mucosal immunity [42]. Thirdly, microorganisms in the gut modulate innate immunity by constantly interacting with innate immune cells such as macrophages, dendritic cells, and innate lymphoid cells. Recognition of microbial-associated molecular patterns (MAMPs) by host pattern-recognition receptors results in the production of inflammatory cytokines and chemokines that are necessary for maintaining the inflammatory threshold. These microbial signals promote the differentiation of tolerogenic dendritic cells and direct macrophage polarization to anti-inflammatory phenotypes and stimulate the production of IL-22 from group 3 innate lymphoid cells (ILCs), thereby enhancing epithelial barrier integrity [37,43].

### 2.1. Pattern-Specific Recognition as a Driver of Immune Activation

The immune system depends on pattern-specific recognition mediated by pattern-recognition receptors to detect conserved molecular patterns of microbes known as microbe-associated molecular patterns (MAMPs). These patterns include microbial proteins like flagellin, bacterial lipopolysaccharides, and peptidoglycans and are unique to the microbe and absent in host tissue; they are considered as a reliable source for the host to distinguish pathogens from self and initiate appropriate immune responses [44,45]. Toll-like receptors (TLRs) are among the best-characterized pattern-recognition receptors expressed on epithelial cells and immune cells such as macrophages and dendritic cells. These receptors recognize specific microbial ligands and activate signaling pathways that trigger transcription factors, such as NF-κB and interferon regulatory factors, to drive the expression of pro-inflammatory cytokines and chemokines [44]. Distinct TLRs recognize specific microbial ligands, as in the case of TLR 4 which is specific for bacterial lipopolysaccharides, while other TLRs detect microbial nucleic acids, lipids, or proteins. Adaptor proteins such as MyD88, are recruited as a result of TLR binding to microbial ligands, initiating downstream signaling pathways such as NF-κB and MAP kinase, resulting in the expression of TNF-α and IL-6, pro-inflammatory cytokines and interferons that collectively promote antimicrobial defense [45]. Thus, TLRs aid in shaping the quality and magnitude of the immune response depending on the structural patterns of microbes encountered.

In addition to membrane-associated TLRs, the cytosolic PRRs are nucleotide-binding oligomerization domain-like receptors (NLRs) that detect microbial products, specifically bacterial peptidoglycan fragments in the intracellular environment. Certain NLRs associate to form multiprotein complexes termed as inflammasomes [46] which activate caspase-1 and aid in maturation and secretion of pro-inflammatory cytokines particularly interleukin-1β (IL-1β) and interleukin-18 (IL-18). These interleukins are known for amplifying local inflammation and shaping downstream adaptive immune responses [47] contributing to pathogen clearance. Together, these pattern-specific recognition mechanisms enable the immune system to distinguish between commensals and potentially harmful pathogens. Instead of inducing uniform inflammation, these receptors elicit context-dependent signaling and are regulated by pathways that promote tolerance to the resident microbiota while invoking potent responses against invading pathogens [43]. Thus, upon initiating an immune response, pattern recognition fine-tunes the quality, magnitude and outcome of immune response, thereby maintaining a balance between host defense and immune homeostasis [45].

### 2.2. Metabolite-Mediated Host Microbiome Crosstalk in Immune Regulation

Microbial metabolites act as key determinants of host immune function. These metabolites serve as chemical mediators that integrate microbial composition, dietary uptake, and host immune pathways, thereby maintaining immune homeostasis and modulating local and systemic inflammation [48,49]. Recent studies on microbiome research have highlighted that differences in metabolites produced as a consequence of microbial diversity and dietary factors have a major impact on immune regulatory pathways. These metabolites modulate signaling through G-protein-coupled receptors, nuclear receptors, and epigenetic mechanisms that guide immune cell differentiation and barrier function [50]. The major classes of gut microbial metabolites include short-chain fatty acids (SCFAs), tryptophan derivatives, secondary bile acid, polyamines, and lipid derivatives. These metabolites serve as one of the mechanisms through which the gut microbiota interacts with the host and regulates the tumor microenvironment (TME). Although these originate from specific microbial metabolic pathways, they function through an interconnected network of signaling pathways that jointly regulate immune homeostasis, epithelial barrier integrity, cellular metabolism, inflammation, and tumor progression. The functional activities of these metabolites are interconnected, with multiple pathways overlapping to influence host response by influencing common molecular targets, such as G-protein-coupled receptors (GPR41, GPR43, and GPR109A), the aryl hydrocarbon receptor (AhR), farnesoid X receptor (FXR), Takeda G-protein-coupled receptor 5 (TGR5), histone deacetylases (HDACs), and inflammatory signaling pathways including NF-κB and STAT3 [12,51]. This coordinated action enables the gut microbiota to promote immune regulation through multiple interconnected mechanisms rather than through the action of a single metabolite (Figure 1, Table 1).

### 2.3. Short-Chain Fatty Acids (SCFAs) and Tryptophan Metabolites

Among the vast repertoire of bioactive metabolites generated by the gut microbiome, SCFAs play a central role in immune crosstalk. Microbial fermentation of dietary fibers by gut bacteria, specifically *Bacteroidetes* and *Firmicutes* species, produces SCFAs such as butyrate, acetate, and propionate that regulate immune cell differentiation through multiple mechanisms [52]. SCFAs strengthen epithelial tight junctions, enhance mucus production, promote differentiation of Foxp3^+^ regulatory T cells, inhibit histone deacetylases, and suppress pro-inflammatory cytokine production through activation of G-protein-coupled receptors such as GPR41, GPR43, and GPR109A [12,13]. These activities collectively reduce chronic inflammation, maintain intestinal barrier function, and limit conditions that induce tumor initiation. However, their biological effects are highly dependent on the immune status, tissue microenvironment, metabolite concentration, and disease stage.

Under normal physiological conditions in the host, SCFAs induce Treg cell differentiation, thereby suppressing the production of pro-inflammatory cytokines. These mechanisms limit chronic inflammation, thereby reducing the risk of inflammation-associated carcinogenesis. Depending on the cellular and metabolic context, SCFAs can also enhance antitumor immunity by promoting the differentiation and function of effector CD8^+^ T cells and Th1 cells, increasing interferon-γ (IFN-γ) production, thereby enhancing the efficacy of immune checkpoint inhibitors [53]. However, in the context of highly immunosuppressive tumor microenvironments, higher SCFA concentrations may predominantly result in expansion of Treg populations, and contribute to immune tolerance, reducing antitumor immunity [54]. Furthermore, this context-dependent activity is depicted in the “butyrate paradox,” wherein butyrate displays either tumor-suppressive or tumor-promoting effects based on the metabolic status of the tumor cells and the host [55]. Altogether, current evidence suggests that the effects of SCFAs cannot be categorized as specifically pro- or anti-inflammatory but should instead be viewed as context-dependent, indicating the interaction between microbial composition, host metabolism, immune cells, and the tumor microenvironment.

Whereas in the case of tryptophan-derived metabolites produced by *Clostridium* spp., *Lactobacillus* spp., and *Peptostreptococcus* spp. activate AhR signaling, resulting in IL-22 secretion, antimicrobial peptide production, epithelial regeneration, and maintenance of mucosal immune tolerance [41,56]. Anti-inflammatory pathways are also induced by receptor activation, contributing to decreased gut inflammation by promoting antioxidant responses by downregulating NF-kB signaling [41]. Despite acting through different receptor systems, SCFAs and tryptophan-derived metabolites perform interconnected functions that regulate epithelial barrier integrity, immune homeostasis, and intestinal health, highlighting the coordinated interactions among microbial metabolites.

### 2.4. Bile Acids, Polyamines, and Lipid-Derived Metabolites

Secondary bile acids represent another group of microbial metabolites that display context-dependent effects on immune regulation and cancer development. Beneficial bacteria such as *Clostridium scindens* convert primary bile acids into secondary bile acids that modulate immune cell differentiation through FXR, TGR5, and VDR signaling [51,57]. Specific bile acid metabolites promote Treg cell differentiation and suppress chronic inflammation, whereas others, when present at high concentrations or in dysbiosis, can induce DNA damage, oxidative stress, and persistent inflammation, resulting in tumor development. Hence, the biological effects of bile acids are influenced not only by their concentration, but also by the tissue microenvironment, microbial composition, and the disease condition. Likewise, microbial polyamines, such as spermidine and spermine, regulate epithelial proliferation, DNA stability, autophagy, and immune cell function. While normal host physiological levels support tissue repair and immune balance, increased polyamine production has been associated with enhanced tumor cell proliferation, angiogenesis, and immune suppression in several cancers.

The major metabolites of lipid metabolism include conjugated linoleic acids (CLAs), omega-3 polyunsaturated fatty acid-derived specialized pro-resolving mediators (SPMs) derived from omega-3 polyunsaturated fatty acids and sphingolipid molecules, all of which regulate innate and adaptive immunity. Conjugated linoleic acids can be obtained from diet or produced by certain strains of bacteria found in the gut such as *Lactobacillus* and *Bifidobacterium* [58]. SPMs are produced as a result of lipoxygenase, cyclooxygenase and cytochrome P450 enzymes on omega-3 fatty acids [59] and aid in resolving inflammation. The metabolites of the sphingolipids include sphingosine-1 phosphate and ceramides that are produced by the host and by certain commensal bacteria present in the gut such as *B. fragilis* and *Lactobacillus* species which help regulate immune homeostasis by modulating host lipid signaling pathways [50,60]. These molecules further influence inflammatory signaling, macrophage phenotypes, dendritic cell maturation, and T cell activation through receptors such as PPARs and TLRs, thereby regulating both innate and adaptive immunity [61,62].

### 2.5. Integrated Effects of Microbial Metabolites in Tumor Progression

Gut microbiota-derived metabolites and their biological effects extend beyond immune regulation, as their combined interactions play a major role in shaping various processes involved in cancer development and progression. Through integrated regulation of inflammatory signaling, epithelial barrier integrity, and host metabolism, these metabolites modulate the initiation and progression of tumors by inducing a tumor permissive or a pro-tumorigenic microenvironment. SCFAs and certain tryptophan-derived metabolites, included under the beneficial metabolites group, maintain intestinal homeostasis by reducing chronic inflammation, promoting epithelial barrier function, and enhancing epithelial barrier function and regulatory immune responses. Conversely, metabolites associated with dysbiosis, such as higher levels of secondary bile acids, polyamines, and specific lipid-derived mediators, can trigger oxidative stress, persistent inflammatory signaling, DNA damage, and aberrant cellular proliferation, thereby promoting tumor initiation and progression [25]. They also modulate metabolic reprogramming by modifying glucose and lipid metabolism, amino acid utilization, mitochondrial function, and epigenetic regulation, thereby aiding tumor cells to adapt within the nutrient-limited and hypoxic TME. Additionally, microbial metabolites influence the balance between antitumor immunity and immune evasion by modulating the differentiation, and functional activity of immune cells, including dendritic cells, macrophages, Treg cells, cytotoxic T lymphocytes, and myeloid-derived suppressor cells [34,61]. The collective effects of these interconnected signaling pathways eventually determine therapeutic responsiveness by regulating antigen presentation, immune checkpoint activation, inflammatory cytokine production, and the efficacy of chemotherapy, radiotherapy, and immune checkpoint inhibitors [35,63]. Accordingly, the comprehensive metabolic pattern generated by the gut microbiota, rather than the action of individual metabolites alone, represents a critical factor determining tumor behavior and treatment outcomes, underscoring the potential of microbiota-targeted interventions as complementary strategies in precision oncology.

**Table 1:** Innate and adaptive immune modulation by gut microbiota.

Microorganism (Species)	Microbial Products/Metabolites	Metabolite/Product Source	Immune System Target	Receptors	Key Cytokines	References
*Akkermansia muciniphila*	SCFAs and membrane proteins	Degradation of mucin	Mucus layer, Dendritic cells and macrophage activation, CD8^+^ T cells response in therapy.	TLR-2	IL-12 and IFN-γ	[64]
*Bacteroides fragilis*	Polysaccharide A (PSA)	Capsular polysaccharide of bacteria	Dendritic cells, macrophages, differentiation of Foxp3^+^ Treg and immune tolerance.	TLR-2	IL-10	[38]
*Lactobacillus plantarum*	Lactic acid, Exopolysaccharides (EPS), SCFAs	Dietary carbohydrates fermentation, secreted products	Natural killer (NK) cells, macrophages, and dendritic cells, Treg and Th1 responses, CD8^+^ T cells response.	TLR-2, NOD2	IL-12, IFN-γ, IL-10, IL-6, TNF-α	[65]
*Clostridium scindens*	Secondary bile acids	Primary bile acid metabolism	Macrophages, NK cells, dendritic cells, colonic Tregs’ expansion.	TGR5, FXR, (GPBAR1), VDR	IL-10, IL-6, TNF-α	[66]
*Bacteroides thetaiotamicron*	Propionate	Fermentation of dietary fiber	Dendritic cells, epithelial cells, Treg cell differentiation.	GPR41, GPR43	IL-10	[12]
*Bifidobacterium* species	Acetate	Fermentation of carbohydrates	Dendritic cell maturation and macrophage activation CD8^+^ T cells’ response.	TLR-2, GPR 43	IL-12, IFN-γ	[63,67]
*Faecalibacterium prausnitzii*	Butyrate	Dietary fiber fermentation	Foxp3^+^ Treg cells, intestinal epithelial cells.	GPR109A, GPR43, HDAC	IL-10, TGF-β, IL-6, TNF-α	[12,40]
*Enterococcus hirae*	Peptidoglycan fragments	Components of cell wall	CD8^+^ T cells, dendritic cells.	NOD2	IFN-γ	[68]
*Escherichia coli* (strains with *pks^+^*)	Lipopolysaccharides (LPS), colibactin	Components of cell wall, secreted by *pks^+^* strains	Macrophage, dendritic cells, T-cell-mediated immunity impaired.	TLR-4 CD14	TNF-α, IL-1β	[69]

Tolerogenic immune programs are promoted by a balanced microbial composition, whereas microbial imbalances (dysbiosis) contribute to inflammatory diseases, immune system dysfunction, and systemic effects. Understanding this metabolic–immune interface is key to deciphering how dysbiosis contributes to immune disorders, autoimmune diseases, and systemic inflammation. Beyond the gut, microbial metabolites influence systemic immunity; for instance, SCFAs can circulate to distant organs such as the liver, lungs, and brain, where they modulate local immune responses and impact disease outcomes, including allergic airway inflammation and neuroinflammation [52].

Disruption of microbial diversity—through antibiotics, poor diet, infection, or chronic stress—can diminish beneficial metabolites, shift immune responses toward pro-inflammatory phenotypes, and increase gut permeability [21]. Dysbiosis has been linked to elevated pro-inflammatory cytokines, heightened susceptibility to cytokine storms in severe infections, and progression of autoimmune diseases and cancers [50].

## 3. Gut Microbiota-Mediated Inflammatory Pathways in Cancer Initiation and Progression

Microorganisms residing in the gut play a significant role in maintaining host equilibrium. Under physiological conditions, beneficial microbes interact with intestinal epithelial and immune cells to maintain immune and mucosal balance. However, alterations in microbial composition can result in increased exposure of host tissues to microbial components and metabolites that activate chronic inflammatory signaling pathways contributing to cancer development. Recent studies suggest that inflammation triggered by microbiota, along with microbial toxins and genotoxic compounds, altogether contributes to the initiation and progression of tumors. These effects are mediated through cytokine signaling, oxidative stress, activation of transcriptional pathways such as NF-κB, and regulation of the tumor microenvironment (TME). Additionally, microbial interactions influence immune monitoring, metabolic reprogramming, and response to cancer therapies.

Chronic inflammation associated with gut microbial imbalance has been linked to a wide range of disorders, including inflammatory bowel disease (IBD), metabolic syndrome, autoimmune conditions, and colorectal cancer. The inflammatory responses induced by dysbiosis can modulate epithelial cell proliferation, disrupt mucosal barrier function, and promote the production of reactive oxygen species and inflammatory mediators, that together result in tissue damage and disease progression [70]. Moreover, persistent inflammatory signaling can shape a microenvironment that favors tumor initiation and progression, leading to a pro-tumorigenic microenvironment.

Furthermore, it has been observed that certain microbial taxa can directly influence immune monitoring and cancer therapy effects by modulating T cell responses, regulating dendritic cell activity, and modifying cytokine signaling pathways. Altogether, these mechanisms emphasize the significance of gut microbiota in modulating inflammation and contributing to the initiation and progression of gastrointestinal and systemic cancers [21,71,72].

### 3.1. Inflammatory Cytokines and Cell Proliferation

The gut microbiome contributes to cancer by modulating inflammatory signaling pathways. Chronic and persistent inflammation is considered to be a hallmark of cancer, initiating tumor progression and metastasis. One of the major pathways involved in mediating inflammatory responses is the NF-κB signaling pathway. NF-κB activation occurs through the Toll-like receptor (TLR) pathway. TLRs include membrane-bound receptors that help in detecting microbial components such as lipopolysaccharides (TLR4), lipoproteins and peptidoglycans (TLR2). Adaptor proteins such as TRIF and MyD88 are recruited on TLR activation, driving the stimulation of downstream pathways, including nuclear factor-kappa B (NF-κB) and mitogen-activated protein kinases (MAPKs). Upon activation, NF-κB leads to the transcription of genes responsible for pro-inflammatory cytokines such as IL-6, TNF-α, and IL-1β, and these aid in establishing a microenvironment that promotes tumor growth and prevents apoptosis. Prolonged stimulation of these pathways leads to sustained inflammation that triggers the production of reactive oxygen species (ROS) that further contribute to oxidative stress and DNA damage, causing genomic instability and enhanced tumor progression [73,74].

Another significant mechanism through which inflammatory mediators are released is through the activation of NLRP3 inflammasome, which is a multiprotein complex having a crucial role in innate immune protection. Microbial components or cellular stress within the immune cells activates the inflammasome which leads to the recruitment of adaptor protein ASC and caspase-1 activation. This process will convert pro-inflammatory cytokines pro-IL-1β and pro-IL-18 into their active forms, which in turn amplifies inflammatory signaling by recruiting immune cells to the inflammation site. Excessive activation of these inflammasomes may result in inflammatory bowel disease and colorectal cancer [75]. Several intracellular signaling pathways, such as STAT3 and MAPK, that regulate genes in cell proliferation and survival are also activated. STAT 3 signaling activation by IL-6 promotes transcription of genes involved in cell cycle progression and cellular survival [76]. The activated NF-κB stimulates the expression of inflammatory mediators and growth-promoting factors that induce cell proliferation. Microbial dysbiosis triggers sustained cytokine production and prolonged stimulation of epithelial cells in the intestinal mucosa, which can result in persistent proliferative signaling and contributes to early tumorigenic changes in the epithelium.

### 3.2. Oxidative Stress and DNA Damage

Another mechanism responsible for chronic inflammation-induced cancer initiation is the production of oxidative stress. Inflammatory responses triggered by microbial dysbiosis can cause the activation of immune cells such as neutrophils and macrophages to produce high levels of reactive oxygen species (ROS) and reactive nitrogen species (RNS). These are produced generally to eliminate pathogens, but persistent production during chronic inflammation can cause DNA damage, genomic instability and can promote tumor progression [70]. Reactive oxygen and nitrogen species can interact directly with cellular DNA, leading to base modifications, DNA double-strand breaks, and chromosomal instability. Inflammatory cytokines produced can also interfere with DNA repair mechanisms, thereby preventing accurate DNA damage repair. Hence, genetic mutations can accumulate specifically in key regulatory genes such as oncogenes and tumor suppressor genes and result in malignancy.

### 3.3. Inhibition of Apoptosis

Apoptotic pathways are disrupted during chronic inflammation, resulting in survival and proliferation of genetically mutated cells. Dysbiosis-induced inflammation produces pro-inflammatory cytokines that activate signaling pathways such as NF-κB and STAT3, thereby stimulating the expression of anti-apoptotic proteins including Bcl-2, Bcl-xL, and Survivin [76]. These proteins inhibit key apoptotic signaling mechanisms and promote cellular survival even when DNA damage has occurred. Some microbial metabolites and toxins may also interfere with apoptosis-related signaling pathways; in the case of colibactin, a genotoxin from *Escherichia coli* may induce DNA alkylation and damage that can disrupt apoptosis, thereby allowing the survival of mutated cells [77]. *Bacteroides fragilis* toxin (BFT) produced by *B. fragilis* and several virulence factors from *Fusobacterium nucleatum* and *Helicobacter pylori* can activate β-catenin, MAPK, and NF-κB pathways, altogether suppressing apoptosis and promoting tumorigenic potential [78,79,80]. Microbial metabolites such as butyrate regulates apoptosis by inhibiting the HDAC enzyme and through epigenetic regulation [81,82]. As a result, epithelial cells that accumulate mutations due to oxidative stress or genomic instability are not eliminated efficiently. The survival and proliferation of these damaged cells increases the risk of tumor initiation and progression within the intestinal tissue.

### 3.4. Epigenetic Modulation

Epigenetic alterations due to chronic inflammation induced by gut microbial imbalance can promote cancer initiation. They alter expression of genes involved in cell proliferation, immune regulation, and tumor suppression, without modifying the DNA sequence. Various epigenetic mechanisms such as histone modification, DNA methylation, and chromatin remodeling are affected by microbial metabolites and inflammatory mediators. Persistent inflammatory signaling may result in abnormal DNA methylation patterns that can suppress the expression of tumor suppressor genes and activate oncogenic pathways [83,84]. Microbial metabolites such as SCFAs, tryptophan-derived compounds, bile acid derivatives, polyamines, and lipid metabolites can modulate immune responses by interacting with host receptors, including G-protein-coupled receptors (GPCRs), that results in the suppression of excessive inflammation by promoting the differentiation of Tregs in maintaining immune tolerance. Histone deacetylase inhibition by SCFAs can influence gene expression and reduce the production of inflammatory cytokines [12,52]. Similarly, tryptophan from diet is metabolized by microorganisms resulting in the generation of active metabolites that can support intestinal barrier integrity and promote mucosal immunity through the activation of aryl hydrocarbon receptor (AhR). Additionally, secondary bile acid derivatives produced from the gut microbial metabolism of bile acids act through host receptors such as Takeda G-protein-coupled receptor 5 (TGR5) and farnesoid X receptor (FXR), thereby regulating inflammatory and metabolic signaling pathways responsible for epithelial barrier function. Microbial imbalances can cause decreased production of beneficial metabolites and an increase in harmful microbial products that can alter epigenetic pathways and may further modify immune signaling, resulting in chronic inflammation [52]. Persistent epigenetic modifications can lead to transformation of normal epithelial cells to cancerous cells.

## 4. Microbiota–Tumor Microenvironment Crosstalk in Cancer Progression

Microbial metabolites and genotoxic compounds act synergistically with chronic inflammation, immune dysregulation and microbial imbalance to create a pro-tumorigenic microenvironment that promotes tumor growth by persistent DNA damage, increased cell survival and enhanced angiogenesis. The tumor microenvironment (TME) is composed of a highly organized network system consisting of cancer cells, stromal components, immune cells and an extracellular matrix [85,86]. Several studies have reported the key role played by the gut microbiome in shaping the TME by interacting with host cells [25,87]; these interactions further influence tumor initiation, progression, immune evasion, and therapeutic responses [34,63] (Figure 2).

### 4.1. Immune Cell Modulation

The gut microbiome has a major influence on immune cell function within the TME. Signals from microbial toxins or components regulate the activity of dendritic cells, T lymphocytes (CD4^+^, CD8^+^) and macrophages. These interactions determine whether the TME develops into pro-tumorigenic or anti-tumorigenic state. Certain microbial species, such as *Fusobacterium nucleatum* and *B. fragilis*, aid in the expansion of Tregs and myeloid-derived suppressor cells that are involved in suppressing antitumor immunity and promoting tumor growth [87], whereas beneficial microbes such as *Akkermansia muciniphila* and *Bifidobacterium longum* can increase cytotoxic T cell (CD8^+^) responses and encourage tumor clearance [63].

### 4.2. Inflammatory Signaling and Cytokine Activation

Inflammation induced by microbiota has a major role in shaping the tumor microenvironment. Pattern-recognition receptors on host cells get activated by certain microbial components, resulting in the release of pro-inflammatory cytokines such as IL-6, IL-1 β, and TNF-α [34,73]. These are responsible for recruiting immune cells such as Tregs and myeloid-derived suppressor cells (MDSCs) to the tumor site and also promote angiogenesis that can enhance tumor cell proliferation and metastasis. Anti-inflammatory cytokines such as TGF-β and IL-10 inhibit effective cytotoxic T cell responses, contributing to immune tolerance. Microbial species in the gut can influence cytokine production by producing metabolites such as SCFAs, which may enhance antitumor immunity by inducing dendritic cell maturation and cytotoxic T cell activation. On the other hand, in a dysbiotic gut, microbial species can enhance tumor-promoting inflammation, facilitating tumor progression [63].

### 4.3. Metabolic Remodeling of the Tumor Microenvironment

One of the hallmarks of cancer progression is the metabolic reprogramming of the TME, facilitating tumor cells to adapt to nutrient limitation, hypoxic conditions, and oxidative stress while promoting proliferation and survival [88]. SCFAs, tryptophan metabolites, secondary bile acids, and polyamines aid in this process by regulating glucose metabolism, amino acid utilization, lipid metabolism, mitochondrial function, and epigenetic modulation in tumor and immune cells [11,35]. This altered metabolism can also influence the function of immune cells such as dendritic cells, macrophages, and T lymphocytes, thereby influencing the balance between antitumor immunity and immune suppression [89]. Moreover, the coordinated interactions between the microbial metabolites and tumor metabolism helps in remodeling the TME by promoting angiogenesis, immune evasion, and resistance to anticancer therapies [34,88]. Apart from these metabolic pathways, mechanisms by which environmental factors, such as nutrient availability, affect microbial behavior and their impact on host immune responses are not fully determined. Recent studies suggest that iron metabolism is linked to gut microbiota-mediated metabolic and immune regulation within the TME [90]. The iron–microbiota–immune crosstalk shapes inflammatory signaling, immune function, and tumor progression, indicating its potential as a therapeutic target in cancer [35,91].

#### 4.3.1. Iron–Microbiota–Immune Crosstalk in Cancer

Iron availability constitutes a significant environmental factor in this context, particularly in the case of microbial secretome dynamics. As an essential micronutrient, iron plays a major role in DNA synthesis, cellular metabolism, and immune function; it also serves as a critical growth factor for many microorganisms. Therefore, iron availability forms an essential point of interaction between the host, microbiota, and tumor cells. Under normal physiological conditions, the host tightly regulates iron through nutritional immunity, which is a defense mechanism that restricts microbial access to free iron. However, in the case of cancer and under persistent inflammatory states, iron homeostasis is disrupted, resulting in accumulation of iron within tissues and the tumor microenvironment (TME) [92]. Disturbances in iron availability can influence microbial communities by favoring the growth of iron-responsive pathogens while suppressing beneficial organisms, thereby contributing to dysbiosis. Many of the bacterial species associated with cancer, such as *F. nucleatum*, *B. fragilis*, certain *E. coli* strains, and *Pseudomonas aeruginosa*, possess siderophores, which are sophisticated iron-acquisition systems that enhance microbial survival and virulence under iron-depleted conditions [93]. These adaptations by microbial species to altered environmental conditions can lead to the synthesis of altered virulence factors and immunomodulatory molecules such as genotoxins, lipopolysaccharides, and inflammatory metabolites. These molecules are recognized by host pattern-recognition receptors (PRRs), resulting in the activation of downstream signaling pathways such as NF-κB, STAT-3, MAPK, and inflammasome-mediated pathways that regulate inflammatory and immune responses. Subsequently, pro-inflammatory cytokines such as IL-6, IL-1β, TNF-α, and IL-17 are released and aid in promoting chronic inflammation, genomic instability, oxidative stress, angiogenesis and tumor cell proliferation. In addition, iron-mediated Fenton reactions promote the formation of reactive oxygen species (ROS) which further trigger DNA damage and mutagenesis, thereby creating a tumor microenvironment that supports tumor initiation and progression. Current studies also indicate that iron availability can have an influence on microbial metabolism, resulting in altered production of metabolites such as SCFAs, polyamines, tryptophan metabolites, and bile acid derivatives. These can modulate immune signaling, inflammatory responses, and cancer cell behavior, thereby influencing the tumor microenvironment and tumor progression [94,95].

Iron metabolism is observed to influence both innate and adaptive immunity, mainly by acting on macrophage polarization, dendritic cell function, T cell activation, and ferroptosis, an iron-dependent form of regulated cell death [96]. Altered iron-handling properties are exhibited by tumor-associated macrophages that are characterized by retention of intracellular iron and the secretion of immunosuppressive mediators that support tumor growth and metastasis. Furthermore, microbial products and their virulence strategies can alter immune cell metabolism and influence ferroptotic sensitivity in both tumor and immune cells [97]. Recent studies suggest that interactions between iron metabolism, microbial metabolites, and ferroptosis pathways regulate immune suppression, hypoxia, angiogenesis, and extracellular matrix reorganization and contribute to TME remodeling [98]. Identification of microbial and iron-related signatures associated with patient outcome and response to immunotherapy has been possible through the advancement of metagenomics, metabolomics, spatial transcriptomics, and single-cell sequencing. These technologies indicate how iron-dependent microbial functions influence immune checkpoint inhibitor efficacy and resistance mechanisms. Exploring the interactions occurring in these interconnected axes can lead to identification of novel therapeutic targets within the tumor microenvironment.

#### 4.3.2. Microbiota–Neuro–Immune–Metabolic Axis

The tumor microenvironment or TME is modulated by a tightly interconnected microbiota–neuro–immune–metabolic axis, comprising microbial signals, neural pathways, immune responses, and metabolic pathways. Persistent interaction of these components regulates tumor progression and treatment outcomes [34]. Gut microbiota generate a plethora of bioactive metabolites and neuroactive compounds, such as tryptophan metabolites, serotonin precursors, SCFAs, gamma-aminobutyric acid (GABA), catecholamines and derivatives of bile acid; these interact with the host through the gut–brain axis involving the enteric nervous system, vagus nerve, and neuroendocrine signaling pathways [99]. These metabolites, particularly the SCFAs and tryptophan-derived metabolites, influence neural signaling by modulation of sympathetic nervous system activity and serotonin synthesis respectively, and help to shape immune responses and inflammation [100]. This can result in release of neurotransmitters such as epinephrine and nor-epinephrine, which in turn activate downstream pathways including NF-KB, STAT3, cAMP, and MAPK pathways that can promote tumor cell proliferation, epithelial–mesenchymal transition, angiogenesis, and inhibition of apoptosis. The neurotransmitters can also suppress cytotoxic CD8^+^ T cells and natural killer (NK) cells while promoting immunosuppressive Treg and myeloid-derived suppressor cells [101]. The metabolic changes induced by these inflammatory and neural signals through various pathways, such as mTOR, STAT3, AMPK, and HIF-1α, result in elevated glutamine metabolism, glycolysis, and lipid biosynthesis, which are cancer biomarkers [88]. An imbalance in microbial flora disrupts the normal functioning of immune responses, as pathogenic microorganisms such as *F. nucleatum*, *B. fragilis*, and *E. coli* will amplify the inflammatory responses and induce pro-tumorigenic signaling through NF-kB, STAT 3, and β-catenin pathways, whereas commensal microbes such as *Fecalibacterium prausnitzii*, *Bifidobacterium longum*, and *Akkermansia muciniphila* enhance immune surveillance and maintain metabolic homeostasis [64]. They also determine therapeutic responses as they enhance the efficacy of immune checkpoint inhibitors by promoting antigen presentation and CD8^+^ T cell activation [102]. Altogether, the microbiota–neuro–immune metabolic axis forms a complex interconnecting network that connects several biological systems to influence tumor behavior and may provide solutions for combinatorial and targeted treatment strategies for cancer.

### 4.4. Regulation of Angiogenesis and Tumor Growth

Microbes residing in the tumor microenvironment can support angiogenesis and tumor progression by eliciting chronic inflammation by activating signaling pathways promoting tumor progression. In the case of *Fusobacterium nucleatum*, a bacteria associated with colorectal cancer, TLRs and NF-kB signaling pathways are activated, leading to enhanced secretion of inflammatory cytokines such as TNF-α, IL-6, and IL-8. These cytokines can induce angiogenesis through vascular endothelial growth factor (VEGF), resulting in tumor growth and progression [87]. *H. pylori*, responsible for inducing gastric carcinogenesis, activates MAPK and STAT3 signaling pathways through *CagA* (Cytotoxin-associated gene A), a major virulence protein produced by *H. pylori.* Activation of these pathways leads to enhanced VEGF expression, blood vessel formation, and epithelial cell proliferation [78,103]. Certain microbial metabolites such as SCFAs can polarize macrophages towards the M2 phenotype, which can promote tumor expansion and metastasis through secretion of angiogenic factors and immunosuppressive cytokines [25]. Inflammation induced by the microbiota can also stimulate neural signaling pathways leading to immune modulation, collectively supporting tumor survival, expansion and resistance to therapies [104]. Altogether, within the tumor microenvironment, there are substantial microbial interactions that can induce a chronically inflammatory and hypoxic environment that is favorable for promoting angiogenesis, tumor growth, and enhanced metastasis.

### 4.5. Microbiota and Immune Evasion

Dysbiosis within the tumor microenvironment plays a major role in tumor immune evasion by promoting immunosuppressive pathways and suppressing antitumor immune responses. Several microorganisms such as *H. pylori*, *F. nucleatum*, *B. fragilis*, and *P. aeruginosa* are known to modulate host immunity to favor tumor survival and progression. As indicated in a study conducted by Sepich-Poore et al., 2021, *F. nucleatum* inhibits CD8^+^ cytotoxic T cell and natural killer (NK) cell activity by interacting with the immune inhibitory receptor TIGI, thereby suppressing immune-mediated tumor clearance and enhancing the progression of colorectal cancer [36]. In the case of gastric carcinogenesis, *Helicobacter pylori* induce gastric inflammation resulting in activation of STAT3 and NF-kB signaling pathways. This activated pathway enhances the expression of immune checkpoint molecules and initiates a tolerogenic microenvironment that supports immune evasion and cancer progression [105]. Microorganisms that produce enterotoxins such as *Enterotoxigenic Bacteroides fragilis* enhance tumor progression by inducing IL-17-mediated inflammation and regulatory T cell expansion. Whereas in the case of opportunistic pathogens such as *P. aeruginosa*, they can affect macrophage polarization towards the M2 immunosuppressive phenotype through iron-regulated virulence factors and quorum sensing molecules [106]. Imbalanced microbial flora can lead to an increased accumulation of MDSCs and secreted cytokines such as IL-10 and TGF-β, resulting in suppression of cytotoxic T cell responses and inhibition of dendritic cell-dependent antigen presentation [104]. Furthermore, persistent inflammation can alter antigen-processing pathways, thereby reducing recognition of tumor-associated antigens by immune cells and enabling tumor cells to escape immune system surveillance. Overall, the immunosuppressive mechanisms induced by microbiota can facilitate prolonged tumor proliferation, metastasis, and anticancer therapy resistance [30,104].

### 4.6. Influence on Cancer Therapy Response

The gut microbiome has been considered as a key player in regulating cancer development, metastasis and therapeutic response. It is known to determine the efficacy of cancer therapies by regulating host immune responses, inflammation, and metabolic pathways. Several studies indicate that these microbiotas can have a major effect on the efficacy of cancer therapies such as chemotherapy, radiotherapy, immunotherapy, and targeted therapy [30,63,72]. The continuous interaction between gut microorganism and the host immune system can influence the tumor microenvironment and governs whether anticancer treatment can lead to effective tumor removal or induce therapeutic resistance. Thus, gut microbiota can serve both as a biomarker and a therapeutic target in cancer treatment strategies.

One such strategy is the use of immune checkpoint inhibitors (ICIs) as part of cancer immunotherapy treatment, wherein these depend on host immune activation to exert the effect and the gut microbiome has been observed to influence patient responsiveness to these therapies. The major checkpoint pathways targeted in cancer therapy are programmed death receptor-1 (PD-1), programmed death ligand-1(PD-L1), and cytotoxic T-lymphocyte-associated protein 4 (CTLA-4). PD-1 receptor present on activated T cells interact with PD-L1 ligand on tumor cells and antigen-presenting cells, resulting in inhibition of T cell proliferation and cytokine production that leads to tumor immune evasion. However, anti-PD-1 and anti-PD-L1 antibodies block this binding which leads to cytotoxic CD8^+^ T cell-mediated killing of cancer cells. Studies have shown that patients responding positively to anti-PD-1therapy observed a higher gut microbial diversity and abundant beneficial bacterial species such as *B. longum*, *A.muciniphila*, *F. prausnitzii*, and *Ruminococcaceae* species. *B. longum* and *A. muciniphila* promote dendritic cell-dependent antigen presentation and IL-12 production, leading to activation of CD8^+^ T cells and enhanced antitumor immune responses [50,107]. Microbial metabolites such as SCFAs, particularly butyrate and propionate, produced by gut commensals such as *F. prausnitzii* and *R. intestinalis*, regulate T cell differentiation, inflammatory signaling, and epithelial barrier integrity, thereby contributing to improved therapeutic efficacy [30].

Chemotherapy efficacy is also influenced by the gut microbiome through several mechanisms such as drug metabolism, immune modulation, and oxidative stress regulation. Chemotherapeutic agents can be metabolized to active or inactive forms by certain bacterial species such as *Gammaproteobacteria*, thereby modulating treatment efficacy by enhancing drug resistance. As observed in a recent study by Xu et al., 2024, *A. muciniphila* and its metabolized form inosine enhanced oxaliplatin responsiveness in gastric cancer by promoting antitumor immunity [108]. Microbial metabolites such as butyrate have been shown to increase apoptosis in colorectal cancer where it sensitizes tumor cells to chemotherapeutic agents by modulating mitochondrial metabolism and reactive oxygen species production [84].

Response to cancer therapies such as radiotherapy and targeted therapies are also influenced by gut microbial composition. Exposure to radiation induces damage to intestinal epithelial barriers and alter microbiome composition leading to dysbiosis and chronic inflammation [109]. Gut commensals aid in protecting against radiation-induced damage to intestinal cells by producing anti-inflammatory metabolites, thus maintaining mucosal integrity. However, colonization of pathogenic bacteria may lead to enhanced production of inflammatory cytokines that can cause oxidative stress and decreased treatment tolerance. Studies on targeted and hormone-based therapies suggest the role of gut microbiota in treatment efficacy as seen in the case of breast cancer, in which estrogen metabolism is regulated partly by a collection of gut microbial genes. These can metabolize estrogens, and dysbiosis can modify estrogen circulation and contribute to hormone therapy resistance [110].

Microorganisms and their metabolites can modulate signaling pathways such as NF-kB, P13K, mTOR, STAT3, and HIF-1α, that are related to cancer-cell survival, angiogenesis, and immune suppression. *B. fragilis*, *F. nucleatum*, have shown to induce poor treatment response and support tumor progression. Chemoresistance in colorectal cancer induced by *F. nucleatum* acts by activating TLR4/NF-κB signaling and autophagy pathways, which reduce chemotherapeutic agent-induced apoptosis [36]. Another pathogen, *B. fragilis*, induces an immunosuppressive tumor microenvironment that weakens antitumor immunity through IL-17-mediated inflammation and expansion of Tregs. Several opportunistic pathogens such as *P. aeruginosa* produce siderophores that can cause iron dysregulation, leading to oxidative stress and activation of pathways that support tumor survival and progression. An imbalanced microbiome can alter antigen presentation, resulting in increased accumulation of MDSCs, and produce immunosuppressive cytokines such as IL-10 and TGF-β which together reduce the effectiveness of chemotherapy and immunotherapy [104]. Thus, these findings suggest that the microbiome indirectly regulates tumor progression and therapeutic outcomes through activation of signaling pathways; a balanced microbiota can support antitumor immunity and help in improving clinical outcomes in cancer immunotherapy treatment. In line with these observations, various microbiome modulation strategies such as dietary interventions, prebiotics, probiotics, and fecal microbiota transplantation (FMT) are currently being extensively studied as novel therapeutic strategies to improve cancer treatment outcomes and in overcoming resistance mechanisms [30].

Table 2 provides an overview of the gut microbiota–tumor microenvironment (TME) signaling axis, indicating key bacterial species, their metabolites, and their impact on host cells. Microbial metabolites and toxins influence immune modulation, inflammatory signaling, metabolic reprogramming, immunosuppression, and therapy resistance.

**Table 2:** Immunomodulatory crosstalk between gut microbiota and tumor microenvironment.

Scheme.	Process	Mechanism of Action	Representative Microbial Species	Target Signaling Pathways	Cancer Type	Effect on Tumor	Therapeutic Implications	References
1	Immune Modulation	Pattern-recognition receptors and metabolites regulate various immune cells (T cells, TAMs, DCs, MDSCs)	*Akkermansia muciniphila*, *Faecalibacterium prausnitzii Fusobacterium nucleatum*, *Bifidobacterium longum Bacteroides fragilis* (ETBF)	NF-κB, MAPK, STAT3, TLR-1, TLR-2	NSCLC, renal cell carcinoma, melanoma, colorectal cancer	Enhanced antitumor immunity	Enhanced immune checkpoint inhibitor efficacy through probiotic and FMT	[34,63,74]
2	Inflammatory mediators	Pro-inflammatory cytokine production (IL-6, IL-17, TNF-α) resulting in prolonged inflammation	*Escherichia coli* (*pks*^+^), *Fusobacterium nucleatum*, ETBF, *Helicobacter pylori*	NF-κB, STAT3, JAK/STAT	Gastric, colorectal, liver cancer	Tumor growth and angiogenesis induction by chronic inflammation	Anti-inflammatory agents and microbiome-targeted therapies	[62,87,111]
3	Metabolic Remodeling	Tumor metabolism and immune differentiation is altered by microbial metabolites such as SCFAs, bile acids, and tryptophan metabolites	*Clostridium* spp., *Bacteroides* spp., *Faecalibacterium prausnitzii*, *Roseburia* spp.	GPCR signaling, inhibition of HDAC, HIF-α, mTOR, AMPK	Hepatocellular, colorectal, pancreatic cancer	Modulates immune responses by reprograming the tumor metabolism.	Microbial metabolism and ferroptosis-targeted anti-cancer therapies	[94,97,112]
4	Angiogenesis and Tumor Growth	Inflammation induced induction of VEGF and pro-angiogenic factors	*Escherichia coli*, *Fusobacterium nucleatum*, *Helicobacter pylori*	NF-κB, HIF-1α, VEGF signaling pathway	Colorectal, gastric, Fibrosarcoma	Tumor progression via enhanced blood vessel formation.	Microbiome modulation and anti-angiogenic therapy	[79,111]
5	Immune Evasion	Cytotoxic T cell suppression and expansion of Tregs and MDSCs	*Prevotella* spp. *Fusobacterium nucleatum*, *Bacteroides fragilis*	TGF-β signaling, PD-1/PD-L1 axis, STAT3	Breast, colorectal, pancreatic cancer	Tumor survival through immune evasion.	Immune checkpoint blockade	[34,74]
6	Response to Therapy	Modulation of immunotherapy, radiation therapy and chemotherapy efficacy	*Bifidobacterium longum*, *Akkermansia muciniphila*, *Ruminococcus* spp., *Enterococcus faecium*	Antigen presentation pathways and IFN-γ signaling, PD-1/PD-L1 axis	Colorectal, renal cell carcinoma, melanoma, NSCLC	Enhanced or reduced therapeutic responses	Personalized microbiome modulation	[63,74,97]

**Figure 2:**
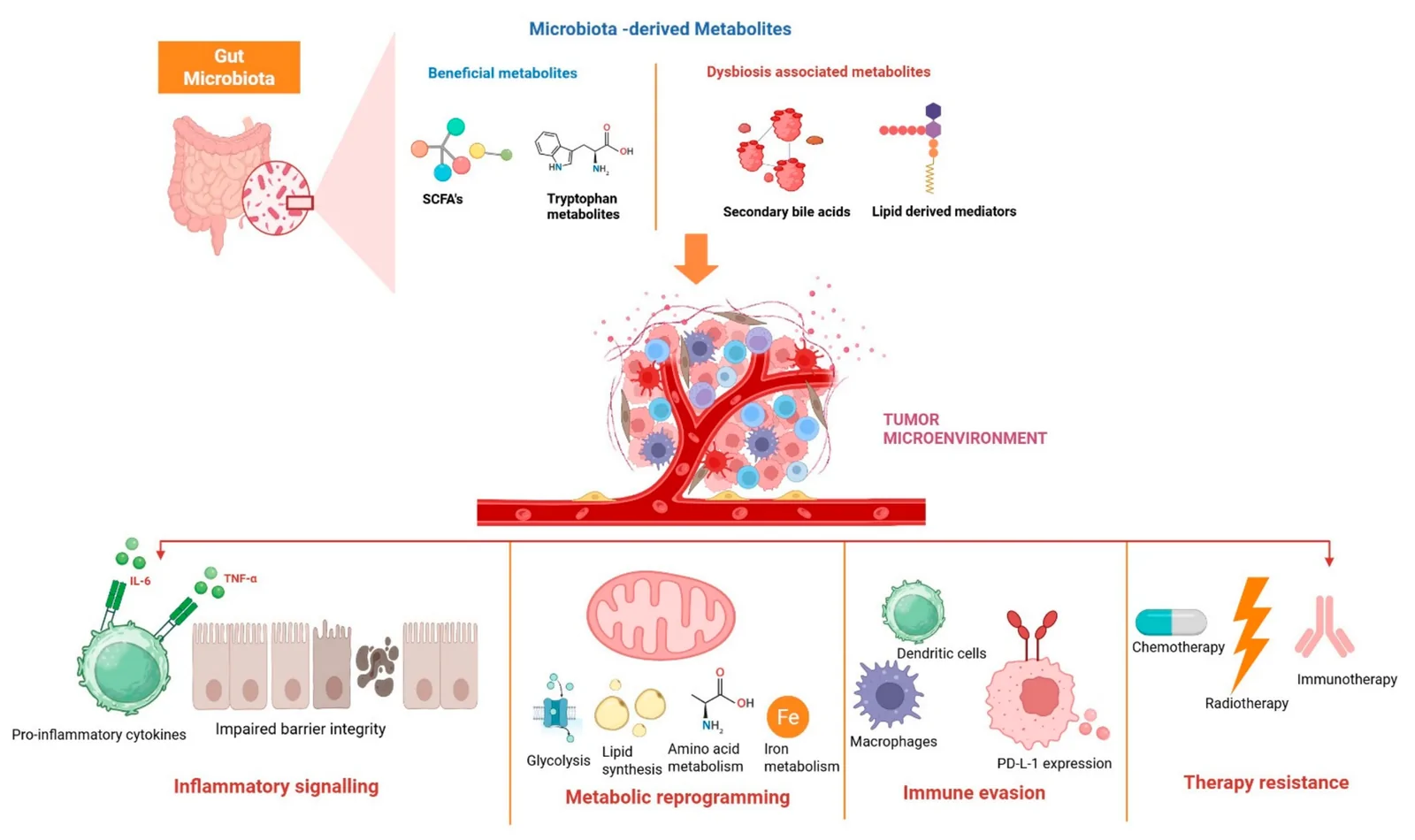
An integrative schematic representation of microbiota-derived metabolites–tumor microenvironment interactions and therapeutic response. Diagram depicting the cross talk between gut microbiota-derived metabolites in immune signaling and inflammation leading to reprogramming the tumor microenvironment. Microbial metabolites and toxins influence immune modulation, inflammatory signaling, TME reprogramming, immunosuppression, evasion and therapy resistance. SCFA—short-chain fatty acids, TNF-α—Tumor necrosis factor alpha, IL-6—Interleukin 6, Fe—Iron, PD-L-1—Programmed death-ligand-1.

## 5. Therapeutic Potential of Targeting the Gut Microbiome

Modulating the gut microbiome has evolved as a major therapeutic approach for improving the clinical efficacy of modern cancer therapies. Beneficial microbial species such as *B. longum*, *A. muciniphila*, *F. prausnitzii*, and *Ruminococcus* spp. have been shown to be associated with improved outcomes in cancer patients receiving treatment with immune checkpoint inhibitors (ICIs) [63,64]. These microbial species use several mechanisms to promote antitumor immunity; primarily, they enhance the maturation of dendritic cells and thus help in effective antigen presentation, and also stimulate CD8^+^ cytotoxic T cell activation, and modulate Th1-polarized immune responses resulting in interferon-γ production. Microbial metabolites form a major component of this regulation; SCFAs such as acetate, butyrate, and propionate, as well as inosine and secondary bile acids act as signaling molecules that shape immune cell metabolism, secretion of cytokines, and epigenetic modifications, thereby modulating the tumor microenvironment toward either immune activation or suppression, depending on microbial balance [113]. Several therapeutic approaches such as dietary interventions, probiotics, prebiotics, synbiotics, and fecal microbiota transplantation (FMT) are recently being explored in both preclinical and clinical studies for cancer treatment.

Recent studies have proven that the uptake of probiotic strains decreases the colonization of pathogenic bacteria, thereby lowering the risks of intestinal infection and inflammation. Next-generation live biotherapeutics and probiotics help in selectively introducing beneficial bacterial strains such as *Bifidobacterium* and *Lactobacillus*. However, current evidence does not give an absolute effect of probiotic use in cancer patients and further clinical studies are essential for exploring the therapeutic effect [114]. In addition to probiotics, prebiotics also have a different effect on gut health. They can inhibit pathogen colonization by mimicking microvillus glycoconjugates, which can interact with bacterial receptors, and the gut can prevent the bacteria from adhering to the host tissue [115]. Colorectal cancer (CRC), which is one of the leading causes of cancer mortality worldwide, is mainly driven by genetic mutations involving oncogenes and tumor suppressor genes. Recent studies have revealed the role of gut microbiota in CRC, particularly in bacterial species such as *F. nucleatum*, which promotes inflammation and tumor progression; *B. fragilis*, that activates Wntβ/-catenin pathway; *E. coli*, which produces colibactin causing DNA damage; *Streptococcus gallolyticus*, associated with tumor microenvironment; and *Enterococcus faecalis* inducing oxidative stress and DNA damage. These bacterial species contribute to carcinogenesis by activating inflammatory pathways such as NF-kB and Wnt signaling pathways. Gut microbiota plays a critical role in CRC and its modulation using prebiotics and probiotics can act as chemo preventive strategy [116].

Postbiotics are soluble byproducts or metabolites secreted by microbiota as a result of biological activities within the host. In some cases, ingestion of postbiotics instead of viable microorganisms is considered as a safer strategy for reducing the adverse effect within the host [117]. Despite the beneficial effects of probiotics and postbiotics on gut health, their capacity to restore the entire diversity and functionality of the gut microbiota remains limited.

Fecal microbiota transplantation (FMT) involves the transfer of an entire fecal microbial ecosystem. FMT is emerging as a promising therapeutic treatment strategy and has shown high efficacy in treating Clostridium difficile infection thereby suggesting its potential in enhancing treatment outcomes and reducing therapy-associated complications [93,118]. In melanoma patients, improved sensitivity to anti-PD-1 therapy is observed through FMT from immunotherapy responders to non-responding patients by introducing beneficial microorganisms and modulating host immunity [102,119]. It aims to modulate systemic immunity, improve the efficacy of cancer drugs, and reduce the toxicities associated with standard treatments. Further clinical studies are essential to confirm its safety and long-term application in cancer therapy.

Synbiotic, which combines probiotic and prebiotics, have been investigated as potential dietary interventions for reducing colorectal cancer risk by modulating gut microbiota and associated metabolic activities. The effect of synbiotic preparation consisting of oligofructose-enriched inulin (SYN1) with the probiotic strains *Lactobacillus rhamnosus* GG and *Bifidobacterium lactis* Bb12 was evaluated in colon cancer patients and polypectomized individuals at high risk of colorectal cancer. The 12-week intervention demonstrated significant alterations in gut microbiota composition, including increased levels of beneficial bacteria such as *Bifidobacterium* and *Lactobacillus* and a reduction in potentially harmful bacteria such as *Clostridium perfringens*. These microbial changes were associated with improved intestinal metabolic activity and reduced exposure to carcinogenic compounds in the colon [120].

Dietary and lifestyle interventions in influencing the gut microbiota have been explored in the case of colorectal cancer metastasis. Evidence from clinical studies suggest that a prebiotic dietary fiber-rich diet and resistant starches shift the microbial flora from inflammatory pathogens toward beneficial organisms like *F. prausnitzii*, which ferment these substrates into SCFAs like butyrate to strengthen the gut-vascular barrier and suppress hepatic inflammation [12]. On the other hand, high dietary consumption of red and processed meats increases the growth of hydrogen-sulfide-producing bacteria and secondary bile acids that degrade this protective barrier. When appropriate dietary changes combined with lifestyle modification such as regular moderate exercise has been practiced, this can lead to increased microbial diversity, along with stress-reduction protocols that reduce cortisol-induced gut permeability, and can starve the tumor microenvironment, converting the host environment into one that is inherently resistant to circulating tumor cells.

Further advanced strategies include engineering bacteria to deliver immunomodulatory molecules directly within the tumor microenvironment, which will offer precise control over immune activation. Studies report that designing specific bacterial strains that can sense tumor specific signals and then release therapeutic drugs such as immune checkpoint inhibitors directly at tumor sites can enhance the treatment outcome with reduced toxicity. This has been observed in the case where an engineered strains of *Salmonella typhimurium* and *E. coli*, selectively target and colonize hypoxic tumor regions and enhance antitumor immunity by producing immune-stimulating factors [121,122]. Such modulation of the microbiome may aid in reducing immune-related adverse events by balancing pro- and anti-inflammatory cytokine networks.

Microbiome research has identified gut microbiota as an important diagnostic biomarker and therapeutic modulator in cancer. Specific microbial signatures, including *B. fragilis* and *F. nucleatum*, are strongly associated with cancer progression, while beneficial microbes such as *Lactobacillus* and *Bifidobacterium* exhibit protective and anti-inflammatory effects. Chemotherapy and immunotherapy significantly alter gut microbial diversity, often reducing beneficial bacteria and increasing pathogenic populations like *E. coli*. Recent studies in cancers such as melanoma, renal cell carcinoma, and non-small-cell lung cancer suggest that microbiome composition can influence treatment response and therapy outcomes. These findings highlight the potential of microbiome profiling in precision oncology for early diagnosis, prediction of therapeutic efficacy, and development of personalized microbiota-based treatment strategies [123].

Studies in colorectal and pancreatic cancer models have shown that intratumoral bacteria can create a pro-inflammatory and immunosuppressive TME. Tumor bacterial burden may also influence immunotherapy response through interactions with the gut microbiome. In head and neck cancer, tumor-associated bacteria were linked to immunosuppression, whereas in other cancers certain bacterial genera may enhance antitumor immunity. For example, *Lactobacillus* has been shown to stimulate antitumor immune responses in melanoma, while *E. coli* was associated with improved immune checkpoint blockade outcomes in lung cancer through cytokine production. These findings suggest that the effects of tumor microbiota on cancer progression and immunotherapy are species- and cancer-type dependent [124].

Immune checkpoint blockade (ICB) is an effective cancer therapy that can provide long-term clinical benefits; however, only a limited number of patients respond successfully. Therefore, identifying reliable biomarkers to predict and improve treatment response is essential. Recent studies highlight the gut microbiome as an important factor influencing ICB efficacy, as microbial composition can be modified through therapeutic interventions to enhance immunotherapy outcomes [125] (Table 3).

Moreover, advances in fecal metagenomics as a tool for identifying early-stage colorectal cancer are in progress. A study by Piccinno et al. has shown that *F. nucleatum*, *P. micra*, and *G. morbillorum* rise significantly by stage I, whereas taxa that gets enriched towards late such as *Slackia exigua* serves as a marker for progression and metastasis [126].

**Table 3:** Beneficial microbial species in successful immune checkpoint inhibitor treatment.

Bacterial Species	Cancer Type	Immunological Mechanism	Reference
*Akkermansia muciniphila*	NSCLC (Lung), RCC (Kidney)	Balances the gut mucus barrier; results in expansion of CCR9^+^ CXCR3^+^ CD4^+^ T helper cells to activate tumor site infiltration.	[64,127]
*Faecalibacterium prausnitzii*	Melanoma	A potent producer of butyrate, that results in up-regulated antigen presentation and CD8^+^ T cell pathways and decreased inflammation.	[84,107]
*Bifidobacterium longum*/*B. breve*	Melanoma, Hepatocellular carcinoma	Trigger TLR4 pathways by releasing bacterial extracellular vesicles, boosting IFN-γ^+^ CD8^+^ T cell responses.	[67,128]
*Ruminococcus bromii*/*R. obum*	Melanoma	Complex starches are degraded and are used to feed other SCFA-producing organisms resulting in decreased cancer progression.	[63,129]
*Collinsella aerofaciens*	Melanoma	Metabolic interaction to alter circulating bile acids, thereby modifying host systemic immune cell trafficking to the tumor.	[127,130]
*Enterococcus faecium*	Melanoma, NSCLC	Releases peptidoglycan-hydrolase enzymes that mimic tumor antigens, thereby priming host T cells to recognize and attack hidden cancer cells.	[130,131]
*Alistipes shahii*	NSCLC (Lung)	Modulation of tumor necrosis factor (TNF) expression by tumor-infiltrating myeloid cells, thereby optimizing them for ICI activation.	[132]

## 6. Discussion

Emerging studies suggest that gut microbial communities and their metabolites play a major role in tumor initiation and progression, immune responses, and treatment efficacy. Nevertheless, the biological effects of microbiota modulation are not consistently beneficial and depend on cancer type, host characteristics, microbial strain, treatment conditions, and disease stage.

### 6.1. Context-Dependent Effects of Microbiota Modulation and Probiotics in Cancer

The effects of probiotics can vary across cancer types and biological contexts. In the case of colorectal cancer, probiotics and synbiotics have indicated beneficial effects, predominantly in reducing gastrointestinal complications and inflammatory responses associated with treatment conditions, though evidence for definitive cancer prevention or improved survival remains limited [133,134]. Likewise, studies conducted in gastric cancer have reported that probiotic supplementation may reduce inflammatory markers, depicting a beneficial effect on the inflammatory microenvironment [135]. However, findings from a melanoma study observed that probiotic supplementation does not consistently enhance antitumor immunity. A study by Spencer et al. reports that melanoma patients undergoing immune checkpoint blockade and have consumed commercially available probiotics had observed lower gut microbial diversity and unfavorable clinical outcomes compared with non-users, whereas increased dietary fiber intake was associated with improved treatment response [129]. In contrast, specific commensal organisms such as *Bifidobacterium* have enhanced anti-PD-L1 efficacy in melanoma models by promoting antitumor T cell responses [67]. These contradictory findings suggest that potential anticancer benefits of probiotics are dependent on the specific strain, cancer type, and clinical condition.

### 6.2. Causality and Correlation in Microbiota–Cancer Interactions

One of the major limitations of the existing literature is the ability to effectively distinguish between causal relationships from associations. Majority of the human microbiome studies have identified differences in microbial composition between cancer patients and healthy individuals, but these observations cannot establish whether this altered microbiome contribute to tumor initiation, may develop as a consequence of tumor progression, dietary changes, inflammation, medication, or cancer treatment. The bidirectional relationship between the gut microbiota and host further complicate the establishment of causality, as microbial composition is influenced by diverse environmental, dietary, and host-specific factors [136]. Stronger evidence is obtained when associations observed in human cohorts are validated by mechanistic experiments, defined microbial consortia, genetic manipulation, microbial transfer studies, or metabolite supplementation. Thus, microbiota and cancer relationships should be evaluated along multiple levels of evidence, ranging from observational associations and mechanistic in vitro studies to causal validation in animal models, and ultimately, through controlled human studies.

### 6.3. Experimental and Clinical Evidence in Microbiota–Cancer Research

Recent evidence regarding the role played by the gut microbiota in cancer has been demonstrated through a combination of in vitro, animal, and human studies; however, the strength of evidence differs substantially across experimental systems. The functional activities of microbial metabolites, such as SCFAs, tryptophan-derived metabolites, polyamines, secondary bile acids, and lipid-derived metabolites, have been predominantly characterized in cell culture and experimental animal models, and have demonstrated their roles in regulating immune cell differentiation, epithelial barrier integrity, inflammatory signaling, metabolic homeostasis, and tumor development [12,13,56]. Nevertheless, cell culture systems cannot reproduce the complex ecological environment of the intestinal microbiome or the systemic interactions between the gut, metabolism, immune system, and TME. Human studies have further indicated associations between altered metabolites and the initiation, progression, or therapeutic response of various cancers, such as colorectal cancer; however, majority of the clinical evidence remains observational and does not establish direct causality [35]. Similarly, the role of gut microbiota in activating inflammatory pathways, such as Toll-like receptor, NF-κB, NLRP3 inflammasome, STAT3, and IL-6/IL-17 signaling, is substantiated by mechanistic evidence from in vitro and murine studies. However, studies conducted in humans have reported correlations between microbial dysbiosis, chronic inflammation, and cancer risk [25,87]. Likewise, the dynamic crosstalk between the tumor microenvironment and the gut microbiota, including immune regulation, metabolic reprogramming, angiogenesis, immune evasion, and therapeutic responsiveness have been established mostly in preclinical models, with supporting clinical evidence from studies exploring microbiome composition and responses to chemotherapy and immune checkpoint inhibitors [34,63,130]. Altogether, these findings provide mechanistic insights into the contribution of the gut microbiota to tumor biology. Further longitudinal cohort studies, standardized multi-omics approaches, and interventional trials are essential for determining causal associations and translating microbiome-based findings into precision oncology approaches.

Although human cohort studies provide greater clinical relevance, they are often observational and thus remain susceptible to potential bias sources and reverse causality. Randomized controlled trials provide stronger evidence for the efficacy of microbiota-directed interventions, although many findings are restricted by small sample sizes, probiotic formulation differences, treatment duration differences, and variation in cancer therapies. Systematic reviews and meta-analyses can strengthen the evidence by combining findings from multiple studies; however, their conclusions are dependent on the methodological quality and comparability of the included studies. Thus, findings from in vitro experiments, animal models, clinical cohorts, randomized trials, and meta-analyses should not be considered comparable levels of evidence.

### 6.4. Translational Challenges from Animal Models to Human Cancer

Clinical translation from experimental models to human cancer is a major challenge. Animal studies have provided significant evidence that microbiota manipulation can influence tumor growth, immune responses, and therapeutic efficacy, but variations among experimental models and humans can considerably affect the reproducibility of these findings. Certain variables including dietary exposure, microbial composition, immune system development, metabolic pathways, and drug responses vary between species [137]. Additionally, outcomes obtained using germ-free or antibiotic-treated animals may not particularly represent the complex microbial environment of patients with cancer. The outcome of FMT is influenced by the differences in donor microbiota, which further can cause variations in human microbiota transfer experiments. Thus, promising findings from animal studies should be considered as translational evidence rather than direct evidence of clinical efficacy. Integration of animal findings with longitudinal human studies and well-controlled clinical trials are required to determine whether mechanisms identified from experiments are clinically relevant.

### 6.5. Challenges in Reproducibility and Methodological Standardization

Another significant drawback is the considerable methodological differences within microbiome research. Variations in sample collection and storage, DNA extraction methods, sequencing platforms, targeted 16S rRNA regions, taxonomic databases, metagenomic workflows, metabolomic techniques, and statistical analyses can lead to considerable differences in the resulting microbiome profiles [138,139,140]. Furthermore, several host characteristics such as dietary habits, medication, age, antibiotic exposure, lifestyle factors, genetics, can contribute to considerable interindividual variation. These factors make it difficult to identify consistent microbial signatures and may explain inconsistent findings across independent cohorts. Thus, standardized methods, comprehensive metadata collection, suitable negative controls, contamination assessment, independent validation, and well-documented reporting are required for improving the reproducibility and comparability of microbiome studies. The development of standardized guidelines and analytical workflows will be important for translating microbiome-derived biomarkers into clinical applications.

Altogether, the current evidence indicates gut microbiota as an important modifier of cancer research but does not completely justify the inclusion of microbiota manipulation as a universally beneficial therapeutic strategy. The variable effects of probiotics, the predominance of correlational evidence in human studies, limitations of animal models, and methodological differences highlight the need for careful interpretation. A multi-level evidence framework connecting mechanistic in vitro studies, causal animal experiments, clinical cohorts, randomized trials, and meta-analyses will be essential for translating microbiome research into clinically reliable treatment approaches, resulting in personalized microbiota-targeted strategies in which particular microbial communities, metabolites, or metabolic functions are selected according to tumor type, host characteristics, and therapeutic context.

## 7. Future Perspectives and Therapeutic Implications

Despite these promising therapeutic advances, successful clinical implementation will require a transition toward precision oncology supported by robust biomarkers and advanced computational approaches. Future research should prioritize the identification and validation of cancer-specific microbial biomarkers using large, ethnically diverse, and longitudinal cohorts while integrating microbiome profiles with complementary genomic, metabolomic, transcriptomic, proteomic, immunological, and clinical datasets. Such multi-omics approaches may substantially improve the sensitivity and specificity of microbiome-based diagnostics and enable the development of companion diagnostics capable of predicting treatment response, identifying patients at risk of immune-related adverse events, and guiding personalized microbiome-directed interventions. In addition, artificial intelligence and machine learning offer significant opportunities to integrate complex biological datasets, identify clinically relevant microbial signatures, predict therapeutic outcomes, and construct digital twin models for individualized treatment planning. However, these approaches require standardized analytical pipelines, larger multicentre datasets, transparent and interpretable algorithms, and rigorous external validation before widespread clinical implementation. Future longitudinal and multicentre clinical trials integrating mechanistic studies, validated biomarkers, and AI-driven predictive models will be essential for translating microbiome research into safe, personalized, and clinically effective cancer therapies [141].

### 7.1. Next-Generation Microbiome Therapeutics

Future investigations should emphasize engineering probiotics with enhanced therapeutic capabilities, enabling them to deliver bioactive molecules such as monoclonal antibodies, RNA-based therapeutics, nanobodies, and immunomodulatory peptides directly within the gastrointestinal tract. In parallel, improving probiotic delivery systems through microencapsulation, nanoencapsulation, hydrogel-based formulations, and pH-responsive coatings will be essential to enhance bacterial survival, intestinal colonization, and long-term engraftment. Standardization of live biotherapeutic products (LBPs) and FMT, including donor selection, manufacturing protocols, dosing strategies, and long-term safety assessment, is equally important for successful clinical translation. Furthermore, optimizing combination strategies with immune checkpoint inhibitors (ICIs), including bowel preparation and microbiome-modulating protocols, represent another important research priority for improving therapeutic efficacy while minimizing adverse effects [142].

### 7.2. Understanding Mechanistic Interactions Within the Microbiota–Tumor–Iron Axis

Recent studies highlight the importance of the microbiota–neuro-immune–metabolic axis in modulating the TME through interconnected immune, neural, and metabolic pathways. Among the several metabolic factors involved, iron metabolism has acquired significance, as altered iron availability within the TME can influence oxidative stress, hypoxia-inducible factor-1α (HIF-1α) stabilization, macrophage polarization, microbial virulence, biofilm formation, and tumor cell proliferation. In addition, iron restriction through nutritional immunity can modify microbial metabolism and immune cell function, affecting T cell activity and therapeutic outcomes. These findings suggest that modulation of the microbiome and tumor metabolism may be considered as promising strategies for improving therapeutic efficacy. Integration of multi-omics approaches with clinical studies may help identify mechanistic links between microbiota, iron homeostasis, metabolism, immunity, and tumor progression. Future studies using advanced experimental models, such as tumor-on-chip models, organoids, single-cell sequencing, and spatial transcriptomics, together with various multi-omics approaches including proteomics, metagenomics, metabolomics, and immunophenotyping, will be required for understanding the functional interactions between immune cells, microbial communities, and metabolic networks within the TME. Through such studies, novel biomarkers and therapeutic targets can be identified to bridge the existing gap between iron homeostasis, microbiome and cancer progression.

## 8. Conclusions

Although significant progress has been made in microbiome research and its application in cancer therapy, several challenges hinder its translation in clinical practice. One of the major difficulties is the considerable variation in microbiome composition among individuals, due to factors such as genetics, age, diet, geographical region, exposure to antibiotics, lifestyle, disease pathology, and immune status. These differences can lead to inconsistent microbial profiles that make it difficult to reliably predict therapeutic responses among patient populations. Additionally, many recent studies do not explore the exact mechanisms by which particular microbial species and their metabolites modulate immune responses, inflammation, tumor progression, and treatment outcomes. Also, the microbial functions differ based on the particular microbial strain present in the tumor microenvironment. TME is a highly dynamic region where microbial metabolites, cytokine signaling, nutrient availability, neural pathways, and changes in host metabolism influence tumor behavior and therapy responsiveness. Reports from various studies indicate that the gut microbiota plays a significant role in modulating antitumor immunity through regulation of immune cells, inflammatory pathways, metabolic signaling and therapeutic responsiveness. Maintaining a balanced gut microbiome can favor the growth of beneficial microbial species, resulting in improved responses to immunotherapy and chemotherapy treatments, whereas dysbiosis may lead to persistent inflammation, immune suppression, therapy resistance, and tumor progression. In conclusion, a deeper understanding of the interactions between microbiota, its metabolism and effects on immunity, and the TME may provide new opportunities for precision oncology and support the development of personalized combinatorial therapeutic approaches for cancer treatment.

## Figures and Tables

**Figure 1:**
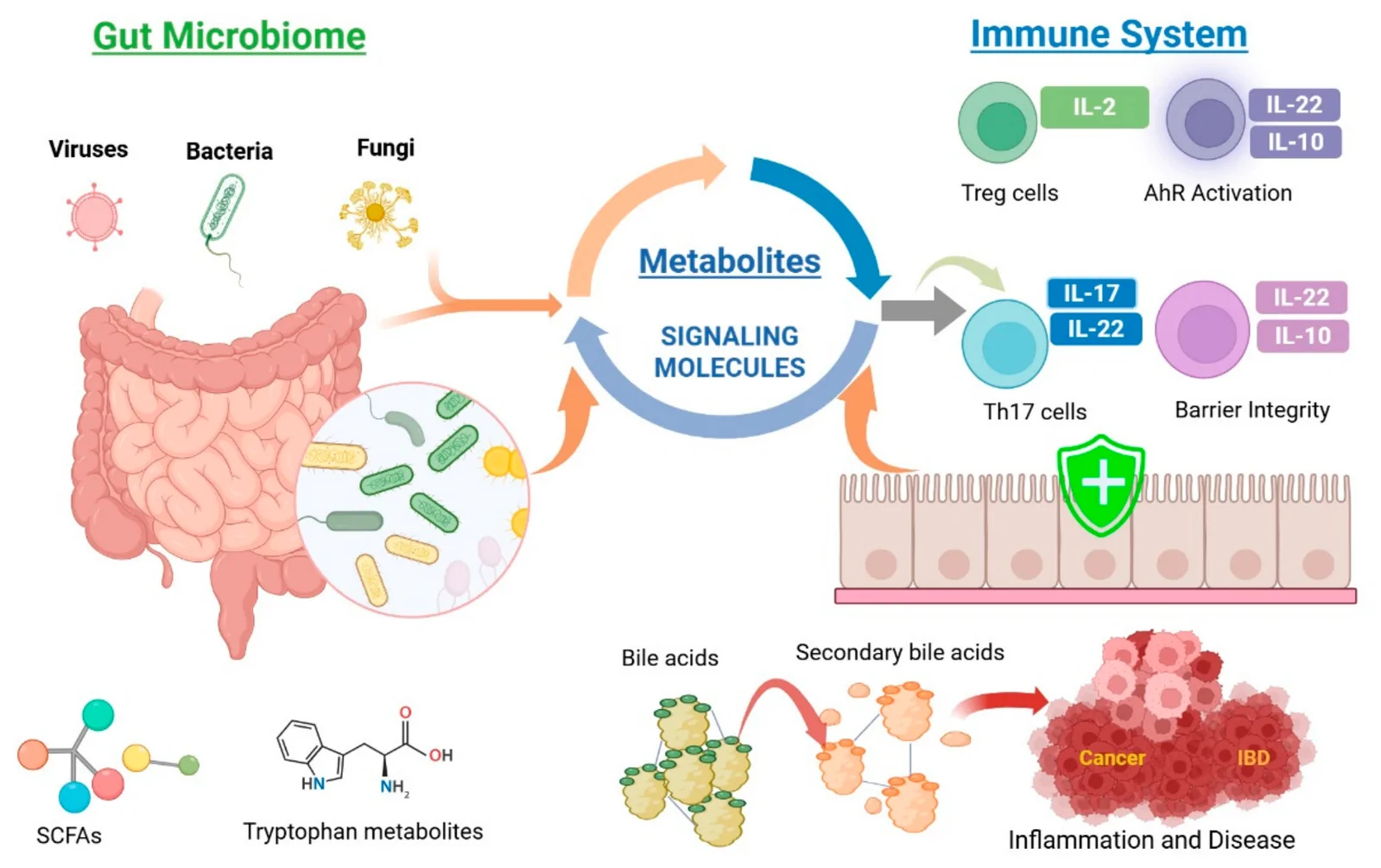
Microbial metabolites and their effects on immune system. An illustration depicting the role played by various microbial metabolites in maintaining immune system homeostasis. Treg cells—regulatory T cells. AhR—Aryl hydrocarbon receptor. Th17—T helper 17 cells. IL—Interleukins, IBD—Inflammatory bowel disease.

## Data Availability

No new data were created or analyzed in this study. Data sharing is not applicable to this article.
